# Next-generation sequencing yields the complete chloroplast genome of *Pleione chunii*, a vulnerable orchid in China

**DOI:** 10.1080/23802359.2019.1640642

**Published:** 2019-07-16

**Authors:** Sha-Sha Wu, Li-Ming Shen, Rui Ling, Zhong-Wu Dai, Zhong-Jian Liu, Si-Ren Lan

**Affiliations:** a Key Laboratory of National Forestry and Grassland Administration for Orchid Conservation and Utilization at College of Landscape Architecture, Fujian Agriculture and Forestry University, Fuzhou, China;; b Fujian Ornamental Plant Germplasm Resources Innovation and Engineering Application Research Center, Fujian Agriculture and Forestry University, Fuzhou, China

**Keywords:** Chloroplast genome, Phylogenetic, Illumina sequencing, *Pleione chunii*, Orchidaceae

## Abstract

*Pleione chunii* is a vulnerable epiphytic orchid with significant ornamental value. Here, we report the first complete chloroplast genome of *P. chunii*. The circular genome was 158,880 bp in length and consisted of a pair of inverted repeats (IR 26,465 bp), which were separated by a large single copy region (LSC 87,259 bp) and a small single copy region (SSC 18,691 bp). It contained 115 unique genes, including 87 protein-coding genes, 38 tRNAs, and eight rRNAs. The maximum-likelihood phylogenetic analysis indicated that *P. chunii* was sister to *P. bulbocodioides* and *P. formosana*.

*Pleione chunii* is an epiphytic or a lithophytic orchid, growing under forests, distribute between 1400 and 2800 m altitude in Northern Guangdong, Guangxi, Guizhou, Hubei, Western Yunnan (Tso 1933; Torelli 2000; Chen et al. 2009). This species has been listed as a vulnerable species in the Red List (IUCN 2018). However, the taxonomy of *P. chunii* and *P. hookeriana* is still in confusion and ambiguity for the lack of extensive sampling and sufficient molecular evidence (Zhang et al. 2018). Comparative analysis of the complete chloroplast (cp) genome of different close species has provided a promising method for the study of phylogeny (Shaw et al. 2014; Jiang et al. 2019). Thus, we aimed to assemble and characterize *P. chunii* cp genomes to provide a better understanding of *P. chunii* and *P. hookeriana*.

In this study, we assembled the complete cp genome of *P. chunii*. The sample of *P. chunii* was collected from Guanyang county, Guangxi province of China (25°53′N, 110°29′E), and voucher specimen deposited at Fujian Agriculture and Forestry University (NO. GXGYchunii01). Total genomic DNA was extracted from fresh leaves using a modified CTAB method (Doyle and Doyle 1987) and sequenced using the Illumina Hiseq 2000 sequencing platform. Raw reads were filtered using NGS QC Toolkit (Patel and Jain 2012). The clean reads were first aligned to *P. bulbocodioides* (Genbank Accession No. KY849819) (Shi et al. 2018) and *P. formosana* (Genbank Accession No. MK361027). Filtered reads were then assembled into contigs in the software CLC Genomics Workbench version 8.0 (CLC Bio, Aarhus, Denmark). After assembled, the obtained scaffolds and contigs were assembled into cp genome by Geneious version 11.1.15 (Kearse et al. 2012) using the algorithm MUMmer. The genome was automatically annotated using DOGMA (Wyman et al. 2004), then adjusted by Geneious version 11.1.15 (Kearse et al. 2012) and submitted to GenBank with accession number MK792342. The cp genome sequence of *P. chunii* is 158,880 bp in length, containing a large single copy (LSC) region of 87,259 bp and a small single copy (SSC) region of 18,691 bp, and two inverted repeat (IR) regions of 26,465 bp. The cp genome encoded 135 genes, of which 115 were unique genes (87 protein-coding genes, 38 tRNAs, and eight rRNAs). Overall GC content of the whole genome is 37.2%, while the corresponding values of the LSC, SSC, and IR regions are 35.0%, 30.3%, and 43.3%, respectively.

To further investigate its phylogenetic position, 85 complete cp genomes of Epidendroideae and two species of Orchidoideae were aligned using HomBlocks pipeline (Bi et al. 2017). RAxML-HPC Black-Box version 8.1.24 (Stamatakis et al. 2008) was used to construct a maximum likelihood (ML) tree with *Ludisia discolor* and *Goodyera fumata* as outgroup. The branch support was computed with 1000 bootstrap replicates. The ML tree analysis indicated that *P. chunii* was sister to *P. bulbocodioides* (KY849819) and *P. formosan*a (MK361027) with 100% bootstrap support (Figure 1).

**Figure 1.**
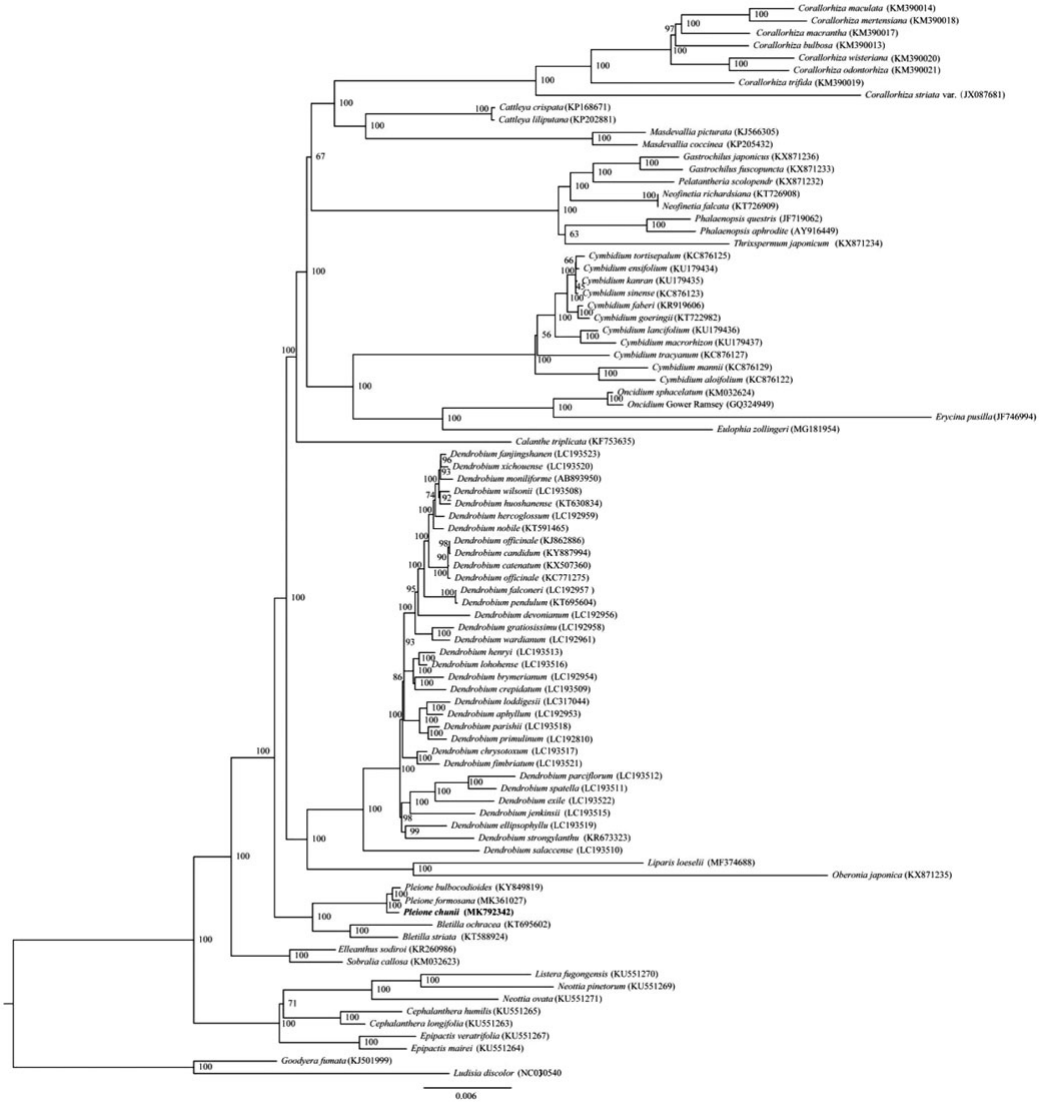
The maximum-likelihood (ML) tree based on 85 species of Epidendroideae, based on whole chloroplast genome sequences, with *Goodyera fumata* and *Ludisia discolor* (Orchidoideae) as outgroup. The bootstrap value based on 1000 replicates is shown on each node, and the position of *Pleione chunii* is shown in bold.
